# Supplementary material for: Drug Prescriptions among Italian and Immigrant Pregnant Women Resident in Italy: A Cross-Sectional Population-Based Study
Source: Int J Environ Res Public Health. 2022 Apr 1;19(7):4186. doi: 10.3390/ijerph19074186 (PMC8998753; doi:10.3390/ijerph19074186)

Supplemental tables and figures

Table S1. Women with at least one prescription of medication by age group and trimester before, during and after pregnancy

	Before Pregnancy						During Pregnancy						After Pregnancy					
	-III		-II		-I		I		II		III		+I		+II		+III	
	n	%	n	%	n	%	n	%	n	%	n	%	n	%	n	%	N	%
**Italians**																		
Age class	116228	32.4	118746	33.1	122697	34.2	186401	52.0	154424	43.1	146049	40.9	166989	46.6	77446	21.6	84640	23.6
≤ 24	6096	27.8	6057	27.6	5971	27.3	11148	50.9	9277	42.3	9070	41.6	9004	41.1	4345	19.8	4652	21.2
25-29	19318	29.2	19810	30.0	20155	30.5	33092	50.1	25965	39.3	25314	38.4	27917	42.2	13285	20.1	14644	22.2
30-34	39225	31.1	40399	32.1	41706	33.1	62779	49.8	50466	40.0	49291	39.2	55243	43.8	26271	20.9	28987	23.0
35-39	36495	34.4	37139	35.0	38626	36.5	56144	53.0	48466	45.7	44252	41.9	52517	49.6	24003	22.7	26113	24.6
≥ 40	15094	39.3	15341	39.9	16239	42.3	23238	60.5	20250	52.7	18122	47.3	22308	58.0	9542	24.8	10244	26.7
**HMPC**																		
Age class	24092	28.0	24600	28.6	24197	28.1	42653	49.5	36053	41.8	38451	44.8	35817	41.6	16946	19.7	17614	20.4
≤ 24	2292	19.9	2361	20.5	2225	19.3	4981	43.3	4183	36.3	4611	40.2	3897	33.9	1736	15.1	1758	15.3
25-29	6424	25.2	6513	25.5	6447	25.3	12111	47.5	9686	38.0	10664	41.9	9346	36.6	4503	17.6	4747	18.6
30-34	7918	29.2	8089	29.8	7926	29.2	13436	49.5	11366	41.9	12249	45.3	11347	41.8	5425	20.0	5693	21.0
35-39	5669	32.9	5797	33.6	5800	33.7	9327	54.1	8235	47.8	8407	49.1	8535	49.5	3958	23.0	4063	23.6
≥ 40	1789	37.8	1840	38.8	1799	38.0	2798	59.0	2583	54.5	2520	53.7	2692	56.8	1324	27.9	1353	28.6

Figure S1. Prevalence of drug use (%) by ATC class during pregnancy

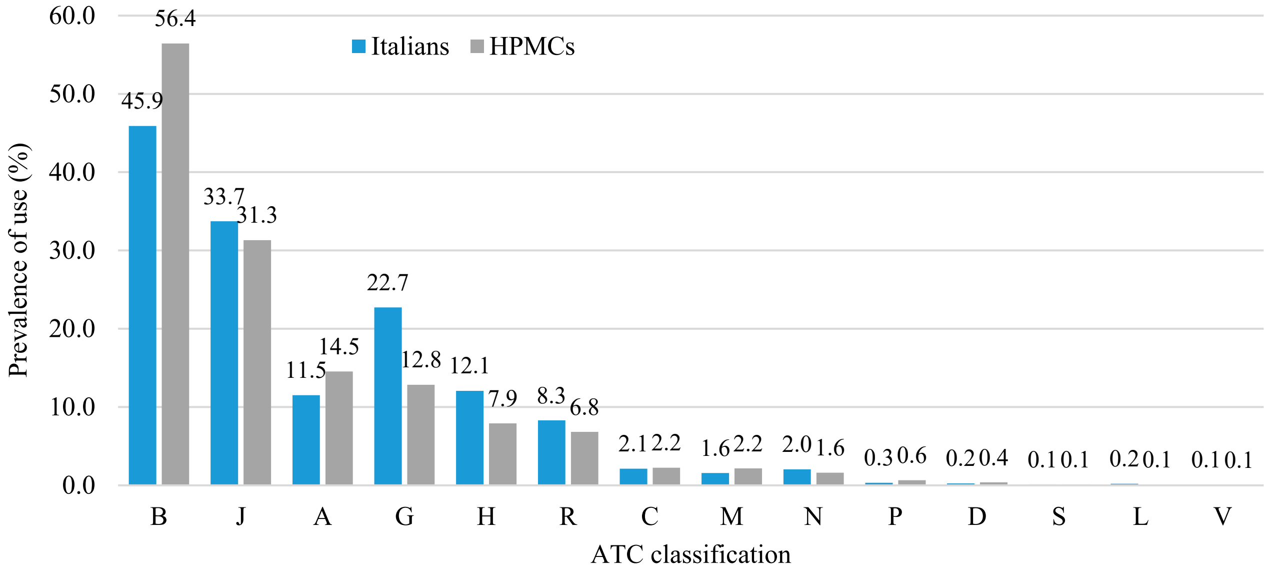

B	Blood and blood-forming organs	R	Respiratory system	D	Dermatologicals
J	Anti-infectives for systemic use	C	Cardiovascular system	S	Sensory organs
A	Alimentary tract and metabolism	M	Musculo-skeletal system	L	Antineoplastic and immunomodulating agents
G	Genito-urinary system and sex hormones	N	Nervous system	V	Various
H	Systemic hormonal preparations, excl. sex hormones and insulins	P	Pesticides		

Figure S2. Prevalence of antibiotic prescription by trimester before, during and after pregnancy

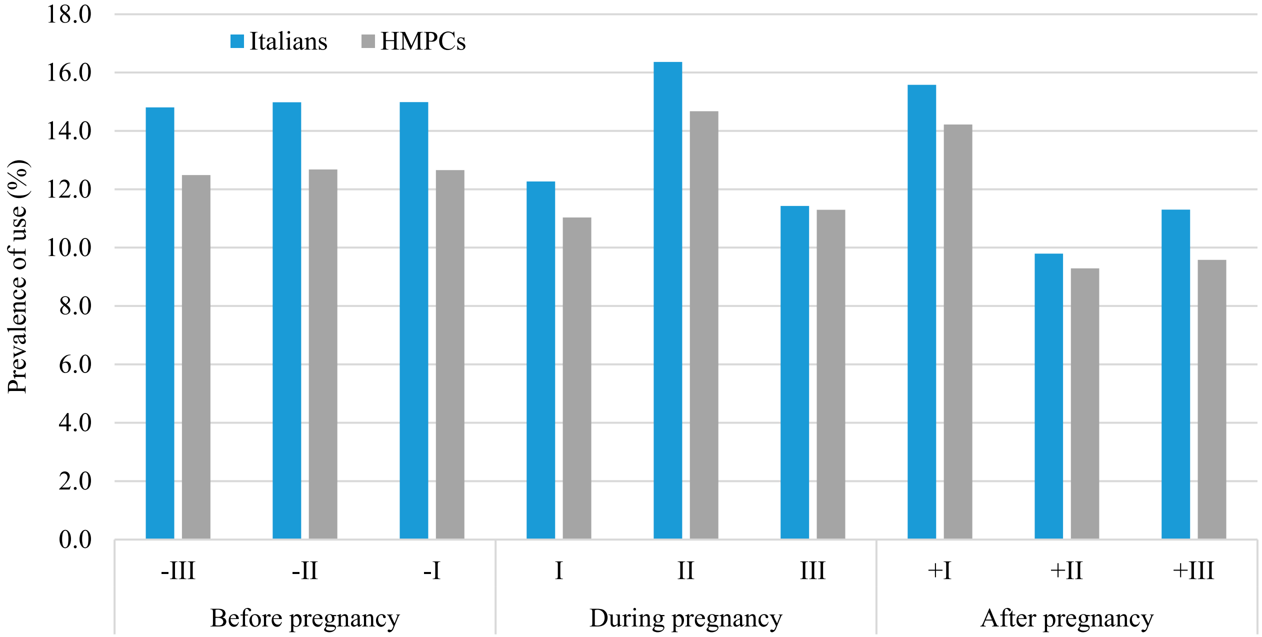

Supplement: Supplementary file 1 [file ijerph-19-04186-s001.zip › ijerph-1619241-supplementary.pdf]
